# Genetic and environmental factors affecting cryptic variations in gene regulatory networks

**DOI:** 10.1186/1471-2148-13-91

**Published:** 2013-04-26

**Authors:** Watal M Iwasaki, Masaki E Tsuda, Masakado Kawata

**Affiliations:** 1 Department of Ecology and Evolution, Graduate School of Life Sciences, Tohoku University, Sendai 980–8578, Japan; 2, RIKEN Advanced Science Institute, 2-1 Wako, Saitama 351-0198, Japan

## Abstract

**Background:**

Cryptic genetic variation (CGV) is considered to facilitate phenotypic evolution by producing visible variations in response to changes in the internal and/or external environment. Several mechanisms enabling the accumulation and release of CGVs have been proposed. In this study, we focused on gene regulatory networks (GRNs) as an important mechanism for producing CGVs, and examined how interactions between GRNs and the environment influence the number of CGVs by using individual-based simulations.

**Results:**

Populations of GRNs were allowed to evolve under various stabilizing selections, and we then measured the number of genetic and phenotypic variations that had arisen. Our results showed that CGVs were not depleted irrespective of the strength of the stabilizing selection for each phenotype, whereas the visible fraction of genetic variation in a population decreased with increasing strength of selection. On the other hand, increasing the number of different environments that individuals encountered within their lifetime (i.e., entailing plastic responses to multiple environments) suppressed the accumulation of CGVs, whereas the GRNs with more genes and interactions were favored in such heterogeneous environments.

**Conclusions:**

Given the findings that the number of CGVs in a population was largely determined by the size (order) of GRNs, we propose that expansion of GRNs and adaptation to novel environments are mutually facilitating and sustainable sources of evolvability and hence the origins of biological diversity and complexity.

## Background

The speed and direction of phenotypic evolution depends on the quantity and quality of genetic variations [1,2], and there have been a number of attempts to quantify such variations [2,3]. Understanding how biological populations generate and maintain genetic variations that contribute to phenotypic variations is one of the most important issues in evolutionary biology. When phenotypic variations are caused by additive genetic variations, evolutionary responses to directional selection are expected to be readily halted because the genetic variation is exhausted in the process of selection [4,5]. However, several experiments involving artificial selection have shown that responses to directional selection can continue for many generations [6,7]. In addition, a population of organisms sometimes shows much larger phenotypic variance when it encounters novel environments compared with that when it lives in normal environments. Standing genetic variations that do not translate into phenotypic differences in the current genetic and environmental background but that can become visible in a different background are called cryptic genetic variations (CGVs). They are considered to contribute to evolution by generating phenotypic diversity in response to changes in the environment and genetic background [8-11].

Various mechanisms are involved in preventing genetic variations from manifesting as phenotypic variations. The ability to retain phenotypes and functions despite internal and external perturbations is called biological robustness [12]. The molecular chaperone called HSP90 is a well-known example of such a mechanism [13,14]. It neutralizes non-synonymous substitutions on DNA sequences by assisting the proper folding of polypeptide chains. Defects in HSP90 or environmental stresses beyond its capacity cause the emergence of hidden variations. A system that contributes to evolvability by hiding and releasing CGVs is referred to as an evolutionary capacitor [13,15,16]. In addition, many genes other than HSP90 are suggested to be involved in stabilizing developmental processes and regulating CGVs [14,17]. Apart form the mechanisms involving a molecular chaperone, redundancy and modularity also produce robustness in organisms [18].

One study [19] proposed a model whereby the release of hidden genetic variation due to a change in the environment or genetic background can be explained by epistasis or genotype–environment interactions. In fact, epistatic interactions were found to be involved in a number of directional selection experiments [20]. Recent studies have also revealed how genes interact with each other during early development (in multi-cellular organisms) and in response to environmental stimuli (in microorganisms) [21-23]. It has recently been suggested that a phenotype is not only the sum of gene effects but also the product of complex interactions among genes [24-26]. Such networks of gene interactions are termed gene regulatory networks (GRNs), and these are considered to play crucial roles in cell differentiation and specific biological functions by modulating the expression of different gene combinations as well as the extent, site, and time of gene expression [27,28]. Epistasis and genotype–environment interactions are generic features of GRNs [29,30], and the biological properties of networks of interacting genes are thus fundamental factors that produce CGVs [29-33].

To understand the nature and the evolution of CGVs, we need to explore the structure and mechanisms of GRNs that accumulate hidden variations and release these variations upon exposure to novel environmental stimuli. We also need to determine how GRNs themselves change during evolutionary processes and to explore the conditions under which evolved GRNs can produce CGVs.

CGVs should be accumulated through population genetic processes, such as mutations, genetic drift, and natural selection. However, most studies of the robustness of GRNs [34,35] have not explicitly assumed gene frequency changes in populations, and in particular, no attempt has been made to examine how CGVs could be accumulated through GRNs that have evolved via population genetic processes even in studies that have discussed the evolution of robustness due to changing gene frequencies [36,37]. To understand the role of GRNs in cryptic variation, we need to construct models that include GRNs that produce different phenotypes when exposed to different environmental stimuli and to examine the number of CGVs acquired through genetic drift and natural selection for each phenotype produced by these GRNs.

This study aimed to show how interactions between GRNs and the environment influence the accumulation and emergence of CGVs. For this purpose, we constructed an individual-based model comprising individuals with plastic GRNs, i.e., GRNs that produce different phenotypes in response to different environmental stimuli. We then determined which factors, e.g., strength of selection, the mode of mutations, the size and properties of GRNs, and the number of environments that the organisms encountered, affected the number of CGVs.

## Methods

### Outline of the model

**Figure 1:**
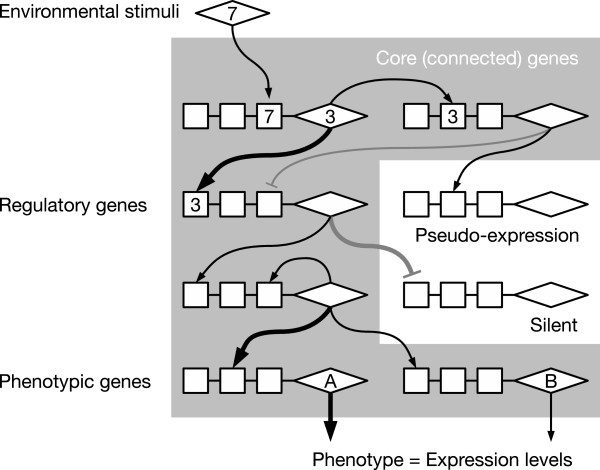
**A schematic example of a gene regulatory network model.** The squares and diamonds represent *cis*- and *trans*-elements, respectively. For simplicity of explanation, only a few of the numbers used for mutual recognition between these elements are shown; however, each element had a number. Black arrow-headed lines and gray bar-headed lines represent transcriptional activation and repression, respectively, and the line weight denotes the intensity of the interaction. Note that different genes would be core genes in different environments.

We constructed an individual-based model similar to that of Tsuda and Kawata [36]. The model population comprised unicellular haploids that reproduced asexually. Individuals had their own genomes, and the genome of an individual determined the structure of that individual’s GRN. The GRN represented a single regulatory module that controlled gene expression in response to environmental stimuli (Figure 1). The phenotype of each individual was defined as the combination of steady-state expression levels of phenotypic genes induced by upstream regulatory genes. The initial GRN of each individual had a random structure. We assumed that the organisms encountered a number of different environments within their lifetime before reproduction and that they plastically produced different phenotypes in response to different environmental stimuli. Physical and biological stimuli such as temperature, light, nutrients, and proteins were assumed to produce *trans*-elements that initiated a gene regulatory cascade. The parameters for GRNs were essentially chosen from values observed in real organisms as well as from values used in other studies [23,36,38-40].

The lifetime fitness of an individual was measured as the geometric mean of the fitness of the phenotypes for different environments, and the fitness for each environment was calculated on the basis of a fitness function for the phenotype in each environment. The individuals reproduced according to their lifetime fitness values. When an offspring was produced, mutations (*cis*- and *trans*-regulatory mutations) were expected to occur. Gene duplications and deletions were not assumed in this model.

A population of individuals was allowed to evolve over a number of generations until a mutation-drift balance was achieved. The genetic and phenotypic diversity and the network characteristics of these were then measured. In addition, phenotypic diversity was measured when the individuals were exposed to novel environmental signals, i.e., signals that differed from those found in the normal environments during the evolution of the population.

### GRNs

The individuals had *V*_*genome*_ (20 by default) genes, which together constituted a GRN. Genes at the downstream terminals, which are known as differentiation gene batteries, have been suggested to represent genes expressed in a cell that fulfill a specific function [23,41]. We assumed that four of the *V*_*genome*_ genes were phenotypic genes that did not have any control over the other genes and that the rest were regulatory genes. Phenotypic genes assigned by us were twice the genes assigned in a previous study [36] to enable us to apply distinct pressures upon phenotypes in different environments. Each gene had a *cis*-regulatory region and a coding region. The coding region of each regulatory gene produced a transcription factor when it was expressed. Each *cis*-regulatory region was composed of *L* (20 by default) *cis*-elements that were potentially recognized by specific transcription factors or signals induced by environmental stimuli. Each *cis*-element and transcription factors had a recognition number (*a*_*cis*_ and *a*_*trans*_, an integral number between 1 and 200). Each transcription factor bound to the *cis*-elements with the corresponding number and induced the expression of the gene. A default value of *L* and a range of recognition numbers were chosen so that the expected number of target genes for a regulatory gene (*L*×*V*_*genome*_/200) was 2.0 [40]. Each *cis*-element and transcription factor also had an interaction coefficient (*v*_*cis*_ and *v*_*trans*_, a real number between −1 and 1) that affected the strength of the transcriptional activation/repression. The absolute value of a coefficient was chosen from uniform random numbers between 0 and 1, and its sign was positive with the fixed probability $\sqrt{A}$ so that the expected proportion of positive edges in a GRN became *A*. We refer to *A* as the positive edge bias and set its default value to 0.6 given the observation that 60% of the edges in the bacteria GRN are positive [39]. In addition to the transcription factors, which were the products of the coding regions, we assumed that each environmental signal was another type of *trans*-element bound to the *cis*-elements, and we set the interaction coefficient to +1.0.

The strength of transcriptional activation/repression was calculated from the interaction coefficients of both the *trans*- and *cis*-element values (*v*_*cis*_, *v*_*trans*_) as well as from the concentration of the *trans*-element (*ρ*_*trans*_). Assuming an additive effect of each element, the intensity of the regulatory input to gene *i*, *x*_*i*_, was calculated as the sum of the effects of all of the *trans*-elements that bound to the *cis*-elements of gene *i*.

(1) $$\;x_{i}=\underset{\text{cis}\;}{\sum }\underset{\;\text{trans}}{\sum }\delta_{\text{cis}\;\text{trans}}|v_{\text{cis}}v_{\text{trans}}|\rho_{\text{trans}}$$

(2) $$\;\delta_{\text{cis}\;\text{trans}}=\left\{\begin{array}{l} 1\;(a_{\text{cis}}=a_{\text{trans}}\cap (v_{\text{cis}}\geq 0\cap v_{\text{trans}}\geq 0))\\ 0\;(a_{\text{cis}}\neq a_{\text{trans}})\\ -1\;(a_{\text{cis}}=a_{\text{trans}}\cap (v_{\text{cis}}<0\cup v_{\text{trans}}<0)) \end{array}\right)$$

The transcription efficiency of a gene is known to show an S-shaped response to the intensity of the input to the gene and can usually be approximated by the Hill function with a Hill coefficient ranging from 1 to 4 [39]. We used a Hill function with a Hill coefficient of 2 as a transcription function. Thus, the transcription rate *β*_*i*_ of the gene *i* was given as follows:

(3) $$\begin{array}{l} \beta_{i}(x_{i})=\left\{\begin{array}{ll} \frac{\beta_{\text{max}}}{1+K_{m}^{2}/x_{i}^{2}}+\beta_{\text{basal}} & \left(x>0\right)\\ \beta_{\text{basal}}\;(x\leq 0) \end{array}\right) \end{array}$$

where *β*_*max*_ and *K*_*m*_ are constants that determine the maximum transcription rate and the threshold against regulatory input; both were set to 0.1 so that the normalized expression level ranged from 0.0 to 1.0 and was balanced with the degradation rate *α* (described below). We assumed that the genes without transcriptional regulation (*x*=0) were also expressed to some extent; therefore, the basal transcription rate *β*_*basal*_ was set to 0.001, which is consistent with the previous study by Tsuda and Kawata[36]. However, this parameter may not have greatly affected the phenotypes observed in this study because we obtained quite similar results when *β*_*basal*_=0.

The concentration of a transcript was determined by the balance of its production and degradation. The rate of change in the concentration *ρ*_*i*_ of the transcripts of gene *i* was given as follows:

(4) $$\frac{d\rho_{i}}{\text{dt}}=\beta_{i}(x_{i})-\alpha \rho_{i}$$

where *α* is a constant that determines the degradation rate of transcripts. This was set to 0.1 because degradation rates are generally slower than transcription rates [42]. The following recurrence equation, which was obtained as an approximation of the previous equation by employing Euler’s method, was used in the simulation:

(5) $$\rho_{i,t+\text{dt}}=\rho_{i,t}+\frac{d\rho_{i}}{\text{dt}}\text{dt}$$

where the time step *dt* was set to 1.0 in the numerical calculations. We regarded the expression of the gene to be constant when the variance of the expression level in 32 recent loops was <10^−6^. These values were determined for computational convenience. Individuals received input signals from environmental stimuli through a signaling pathway that functioned via *trans*-elements that activated the expression of genes with the corresponding *cis*-elements. In this model, each *trans*-element from an environmental stimulus also had a random *trans*-number (*a*_*trans*_, an integral number between 1 and 200, the same as the transcription factors described above) and a constant interaction coefficient (*v*_*trans*_, +1.0).

All the mutations in our simulation were point mutations on *cis*- or *trans*-elements, and they could be categorized into four types of changes: *cis*-number, *cis*-coefficient, *trans*-number, and *trans*-coefficient. Each type had the same mutation rate (*μ*), and the default value was set to 10^−4^ per individual per generation, which was in balance with the population size *N* (described below).

### Phenotype and fitness

The level of gene expression reached a stable state after the GRN was activated by environmental signals. The phenotype of an individual was defined as the combination of the stable-state expression levels of four phenotypic genes. The phenotypic value of an individual was represented by a point in four-dimensional space with four axes, each of which denoted the expression level of a phenotypic gene. The individuals whose expression levels were very low (<0.01) or did not reach stable states after 5,000 loops of interaction were assumed to be dead. These values were determined for computational convenience.

The fitness of an individual was defined as a decreasing function of the distance from the optimal expression levels, which differed in different environments. The fitness of each individual *i* in environment *j* was given as follows:

(6) $$\Phi_{i,j}=\exp(-\frac{d_{i,j}^{2}}{2\sigma^{2}})$$

where *d*_*i*,*j*_ is the Euclidean distance from the optimum defined by the environment *j* and the phenotypic value of the individual *i*, and *σ* is the parameter that determined the breadth of the foot of the fitness landscape. For simplicity of explanation, we used *S*=−*ln*2*σ*^2^ to represent the strength of selection and set it to 3.0 by default. When *S* was larger, the selection gradient became steeper and small changes in phenotypic values were subjected to stronger selective pressure, i.e., the mutations that produced phenotypic variations were supposed to be purged more quickly from the population.

An environmental stimulus induced signals as *trans*-elements in all the individuals in a population, and the corresponding optimal value of the phenotypes was given in the environment. Individuals experienced *H* (two by default) different environments during their lifetime. The lifetime fitness of an individual was given by the geometric mean of the fitness in all of the environments. We assumed non-overlapping generations and a constant population size, *N* (100,000 by default). Therefore, the default value of *N*×*μ* was 40 per generation, which is consistent with the estimated values for bacteria and yeast [38]. The expected number of offspring, *W*_*i*_, of the individual *i* whose lifetime fitness was *Φ*_*i*_ was calculated as follows:

(7) $$W_{i}=N\frac{\Phi_{i}}{\sum_{k}^{N}\Phi_{k}}$$

### Simulation procedure

**Figure 2:**
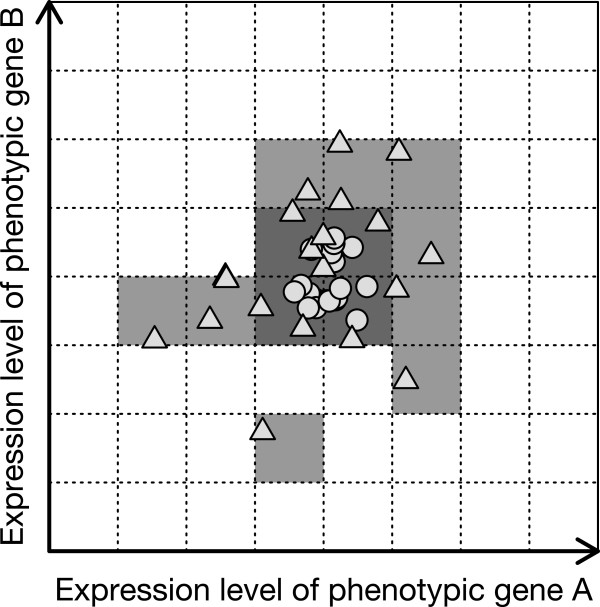
**A schematic example of evaluating phenotypic diversity.** For simplicity, a two-dimensional space is shown; however, the phenotypic space used in our analysis was four-dimensional. The two axes denote the expression levels of phenotypic genes 1 and 2, respectively. The phenotype of an individual gives the coordinate of a point in this space. The space was divided into grid cells. The phenotypic diversity of a population was defined as the number of cells in which the individuals in the population were found. The circles and triangles denote the individuals’ phenotypes in normal and novel environments, respectively. In this example, the phenotypic diversity of the population was four in the normal environment (dark gray cells) and 13 in the novel environment (light gray cells and some dark gray cells). In this case, the number of cryptic genetic variations was calculated to be nine (i.e., 13−4).

A population of individuals with random genomes was created and allowed to evolve under normal conditions. The normal conditions consisted of *H* different environments, and all the individuals experienced all the *H* environments during their lifetime. Each environment had different stimuli-inducing signals and corresponding optimal phenotypic values. Individuals could evolve to adapt to the normal conditions and increase their lifetime fitness over 20,000 generations. When the lifetime fitness of some individuals in the population exceeded 0.99, a clone population was created by copying the individual with the highest fitness. Thus, the new initial population harbored only the individuals that were well adapted to the normal conditions. This clone population was then allowed to evolve under the same normal conditions for 20,000 generations. The number of standing genetic variations (*G*) in the population could reach a stable state during this period. This was measured as the number of polymorphic GRNs in the population, i.e., genetic variations that caused no changes in gene interactions were not included in the count. To evaluate the phenotypic diversity of a population, we divided the phenotypic space into grid cells (Figure 2). The phenotype of each individual could be placed in one of these cells. The number of cells in which the phenotypic values of the individuals in the population were placed was defined as the phenotypic diversity of the population. At the end of the simulation, the phenotypic diversity of the population in one of the environments under normal conditions (*P*_*normal*_) was measured. Thereafter, the population was subjected to a novel environment with a novel stimulus and the phenotypic diversity (*P*_*novel*_) was again measured. If *P*_*novel*_ was larger than *P*_*normal*_, CGVs were expected to present. The number of CGVs (*P*_*cryptic*_) was thus measured as *P*_*novel*_−*P*_*normal*_.

### Analysis of factors affecting the number of cryptic variations

To determine which factors associated with the GRNs affected the number of CGVs, the following network properties were analyzed: network order, network size, density, average clustering coefficient, number of self-loops, and degree assortativity. The methods used for calculating these properties are described in the next section. In addition, the number of standing genetic variations in a population (*G*, previously defined) was also included as a properties that affected the number of CGVs. These network properties could not be fixed as parameter values in the simulations because they were randomly varied at the initial states and were changed by evolution during the simulation runs. Thus, they varied among the simulations even with the same parameter values. The properties were measured as the average values of all the individuals in a population and 120 replicated populations under the same parameter set (default parameter set, Table 1) were used for the analysis. All statistical analyses were performed using R 2.15.2 software [43]. We used a generalized linear model (GLM) for which the response and explanatory variables were the number of CGVs (*P*_*cryptic*_) and GRN features, respectively. We extracted the best predictors with nested model comparisons by calculating the Akaike Information Criterion (AIC); explanatory variables were removed and added to the models to determine the set of predictors that yielded the lowest AIC.

**Table 1:** Range and default parameters used in the simulations

**Symbol**	**Variable**	**Min.**	**Max.**	**Default**
*μ*	Mutation rate [gene^−1^ generation^−1^]	5.0×10^−5^	1.5×10^−4^	1.0×10^−4^
*N*	Population size	50,000	150,000	100,000
*V*_*genome*_	Number of genes	10	30	20
*L*	Number of *cis*-regulatory elements	10	30	20
*A*	Positive edge bias	0.3	0.6	0.9
*H*	Number of environments	1	6	2
*S*	Strength of selection	1.0	6.0	3.0

The number of genes (*V*_*genome*_), the number of *cis*-regulatory elements per gene (*L*), and positive edge ratio (*A*) were set as the initial parameter values given their effects on network properties such as order, weighted size, and density. The population size (*N*), mutation rate (*μ*), strength of stabilizing selection (*S*), and number of environments (*H*) were also set as the initial parameter values. The effects of each of these properties on the number of CGVs were examined by modifying them. The values for these parameters are shown in Table 1. Simulations were conducted 20 times for each parameter. The number of standing genetic variations (*G*), visible phenotypic variations (*P*_*normal*_), and cryptic variations (*P*_*cryptic*_) was measured after the simulations (see the previous subsection for their definitions). We obtained regression coefficients from a GLM in which *G*, *P*_*normal*_, or *P*_*cryptic*_ was the response variable, and each of the parameters was an explanatory variable.

### Network properties

The structural properties of biological networks are considered to be responsible for biological processes such as environmental response and cell differentiation [44,45]. The following network properties were measured using NetworkX 1.7, a Python language software package [46].

#### Network order

Gene duplication was not assumed in our model, and the number of genes that an individual had was determined by a parameter (*V*_*genome*_). However, not all the genes were responsible for phenotypic expression in our model because how the genes interacted with each other depended on the randomly variable *cis*-regulatory elements of the genes. Thus, we defined core genes as the genes in a GRN that were connected to phenotypic genes and the network order (*V*_*core*_) as the number of core genes.

#### Network size and density

Genes and the regulatory interactions among them were represented as nodes and edges, respectively. The network size (*E*) was defined as the number of edges among the core genes. Similarly, the weighted size of the network (*E*_*weighted*_) was defined as the sum of the weight of all the edges where the weight of an edge was the strength of the regulatory interaction. The density of a network denotes the ratio of the number of existing edges to the maximum possible number of edges among the core genes and was calculated as follows:

(8) $$\text{density}=\frac{E}{V_{\text{core}}^{2}}$$

If the two genes showed a regulatory interaction, we regarded them as being neighbors. The number of neighbors of gene *i* was defined as degree *k*_*i*_. In our model, the expected density values were dependent on the number of genes and *cis*-regulatory elements of each gene.

#### Average clustering coefficient

The clustering coefficient *C*_*i*_ of gene *i* was given as the ratio of the actual number of edges *e*_*i*_ between the neighbors relative to the maximal number. We calculated the average clustering coefficient $\bar{C}$ of all the core genes as a network property as follows:

(9) $$C_{i}=\frac{2e_{i}}{k_{i}(k_{i}-1)}$$

(10) $$\bar{C}=\frac{1}{V_{\text{core}}}\underset{i\in \text{core}}{\sum }C_{i}$$

The average clustering coefficient of a network tends to increase when the network has more locally dense compartments, i.e., clusters. Cancerous networks of oncogenes in humans are known to form a giant, highly clustered component in the whole signaling network [47].

#### Number of self-loops

When a gene product regulates its own expression, it is a form of autoregulation and is denoted by a loop in a network called a self-loop. It is known that negative feedback stabilizes its expression level and shortens the response time, while positive feedback delays the response and results in bistability [39]. A mutagenesis experiment with yeast confirmed that genes with negative feedback loops are mutationally robust [48].

#### Degree assortativity

A network is said to be assortative (or disassortative) when a high-degree node tends to connect with nodes of high (low) degree. Protein interactions and GRNs in yeast and the neural network in nematodes are known to be disassortative [49,50]. Assortative networks have higher thresholds for perturbation to change the outcome and are thus more robust, whereas disassortative networks are thought to be resilient against small perturbations [49,51]. Assortativity was calculated as the Pearson correlation coefficient of the degree of nodes at the ends of the *i*th edge in the core gene network as follows:

(11) $$\text{assortativity}=\frac{4E\sum_{i\in \text{core}}j_{i}k_{i}-{\left[\sum_{i\in \text{core}}j_{i}+k_{i}\right]}^{2}}{2E\sum_{i\in \text{core}}j_{i}^{2}+k_{i}^{2}-{\left[\sum_{i\in \text{core}}i_{j}+k_{j}\right]}^{2}}$$

#### Positive edge ratio

Whether the expression of a gene was activated or repressed by the binding of a transcription factor was determined by the signs of their interaction coefficients. The expected value of the ratio of positive edges to negative edges was dependent on the bias parameter *A*: however, it randomly varied during the initial state and was changed by the evolution during the simulation. We measured the realized ratio of positive edges to negative edges at the end of the simulation.

### Analysis of factors affecting network properties

Network properties evolved during the simulations. We examined the effects of the number of genes, number of *cis*-regulatory elements, mutation rate, population size, strength of stabilizing selection, and number of environments on the properties of the evolved networks by altering these parameter values. Simulations were conducted 20 times for each parameter, and the average values of the network properties were measured thereafter. A GLM was then created with each parameter as a response variable and the average network property as an explanatory variable.

## Results

### Cryptic variations in GRNs

**Figure 3:**
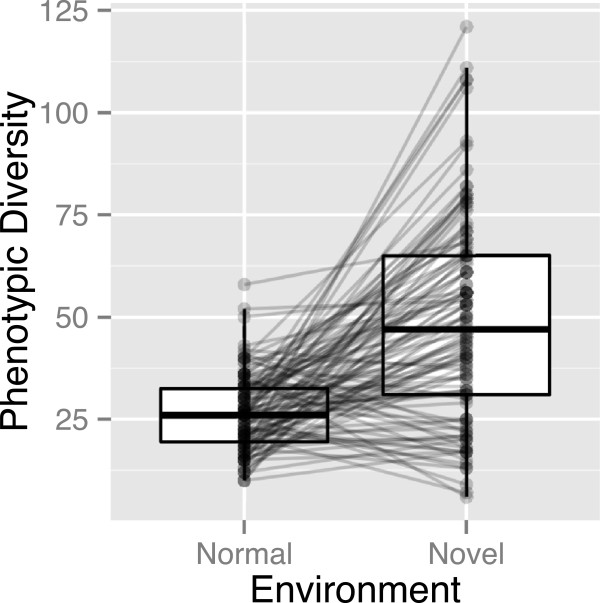
**Comparison of phenotypic diversity between normal and novel environments.***P*_*novel*_ was significantly larger than *P*_*normal*_ (Wilcoxon signed rank test, *V*=5825, *P*=4.298×10^−14^).

In the default parameter set (Table 1), significantly more phenotypic variations appeared in response to a novel environmental change (*P*_*novel*_) compared with variations observed under the normal condition (*P*_*normal*_) during stabilizing selection over 20,000 generations (Figure 3; *V*=5825, *P*=4.298×10^−14^). In the present simulations, phenotypic differences were caused by at least one genetic difference in the GRNs; therefore, the phenotypic variations were partly due to the total genetic variation. Thus, *P*_*novel*_>*P*_*normal*_ meant that genetic variations that had no influence on phenotypic variations when responding to normal environmental stimuli were accumulated and these produced phenotypic variations when responding to novel environmental stimuli in the population of GRNs during stabilizing selection (i.e., *P*_*cryptic*_=*P*_*novel*_−*P*_*normal*_ could be considered indicative of CGVs).

### The effect of GRN properties on the accumulation of cryptic variations

**Figure 4:**
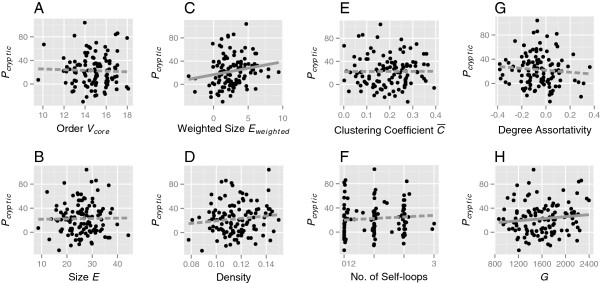
**Relationships between cryptic variations and network properties under the default parameter condition.** The weighted size of the network, genetic variations, and degree assortativity were selected as the variables that explained the number of cryptic variations using GLM model selection. Regression lines were drawn on the basis of simple linear regression analysis.

The number of CGVs in each of populations varied greatly, even with the same parameter sets (Figure 3). This may be partly because the properties of the initial and subsequent GRNs varied owing to random factors. We thus examined the effects of the properties of variable GRNs in the same default parameter set on the number of CGVs (*P*_*cryptic*_) were examined. Figure 4 shows the relationships between the number of CGVs and the properties of the GRNs (the network order, size, weighted size, density, clustering coefficient, number of self-loops, degree assortativity, and number of genetic variations). Of these network properties, only weighted size and genetic variations were selected as variables that explained the number of CGVs. The number of CGVs increased along with the increases in the weighted size of the GRNs (*t*=2.463, *P*=0.0153) and in the number of genetic polymorphisms in the populations (*t*=1.908, *P*=0.0590). The model selection statistics for the effect of GRN features on the number of CGVs (*P*_*cryptic*_) are shown in Additional file 1: (Table S1).

### Effects of population size, mutation rate, number of genes, number of *cis*-regulatory elements, and positive edge bias on cryptic variations

**Figure 5:**
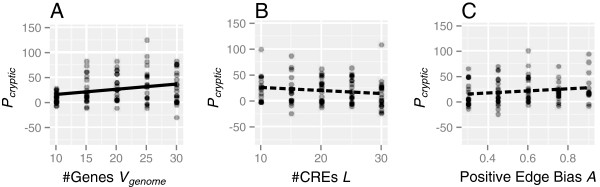
**Relationships between cryptic variations and altered GRN parameters.** Simulations were repeated 20 times for each parameter value, and each dot represents a simulation run. Regression lines were drawn on the basis of the linear regression statistics shown in Table 2; solid and dashed lines denote significant and non-significant correlations, respectively.

The effects of the population size (*N*), mutation rates (*μ*), number of genes (*V*_*genome*_), number of *cis*-elements (*L*), and positive edge bias (*A*) on genetic variations maintained in the populations (*G*), visible phenotypic variation (*P*_*normal*_), and accumulation of CGVs (*P*_*cryptic*_) were examined by altering these parameter values. Table 2 shows the regression coefficients and statistical significance of these effects after adjustment for multiple comparisons [52]. *G*, *P*_*normal*_, and *P*_*cryptic*_ in the populations were significantly increased with increases in *N*, *μ*, and *V*_*genome*_ (Figure 5A). *G* decreased with increasing numbers of *cis*-elements (*L*) under a constant mutation rate per individual, whereas visible and cryptic phenotypic variations (*P*_*normal*_ and *P*_*cryptic*_) were not affected (Figure 5B). On the other hand, positive edge bias (*A*) caused a slight increase in the number of CGVs (*P*_*cryptic*_; Figure 5C) despite the reduced number of genetic polymorphisms (*G*).

**Table 2:** The effects of each parameter on population variation

**Response variable**	**Explanatory variable**	**Estimate**	**Observed P**	**Rank of P**	**Adjusted*α***
*G*	*N*	0.0185	5.479×10^−25^^∗^	4	0.00952
*P*_*normal*_	*N*	9.921×10^−5^	0.000401^∗^	11	0.0262
*P*_*cryptic*_	*N*	0.000249	0.00253^∗^	12	0.0286
*G*	*μ*	1.389×10^7^	1.608×10^−22^^∗^	5	0.0119
*P*_*normal*_	*μ*	1.706×10^5^	2.936×10^−9^^∗^	8	0.019
*P*_*cryptic*_	*μ*	1.421×10^5^	0.0327^∗^	15	0.0357
*G*	*V*_*genome*_	117	1.371×10^−37^^∗^	1	0.00238
*P*_*normal*_	*V*_*genome*_	0.914	4.877×10^−11^^∗^	7	0.0167
*P*_*cryptic*_	*V*_*genome*_	1.04	0.0132^∗^	14	0.0333
*G*	*L*	−21.1	5.302×10^−5^^∗^	10	0.0238
*P*_*normal*_	*L*	0.23	0.0959	17	0.0405
*P*_*cryptic*_	*L*	−0.589	0.15	19	0.0452
*G*	*A*	−720	1.900×10^−5^^∗^	9	0.0214
*P*_*normal*_	*A*	5.52	0.154	20	0.0476
*P*_*cryptic*_	*A*	20.6	0.124	18	0.0429
*G*	*S*	−294	2.105×10^−28^^∗^	3	0.00714
*P*_*normal*_	*S*	−12.6	5.995×10^−32^^∗^	2	0.00476
*P*_*cryptic*_	*S*	0.494	0.792	21	0.05
*G*	*H*	−239	3.709×10^−22^^∗^	6	0.0143
*P*_*normal*_	*H*	1.05	0.0443	16	0.0381
*P*_*cryptic*_	*H*	−4.47	0.00687^∗^	13	0.031

The effects of population size (*N*), mutation rate (*μ*), number of genes (*V*_*genome*_), number of *cis*-elements (*L*), positive edge bias (*A*), strength of selection (*S*), and number of environments (*H*) on genetic variations maintained in the populations (*G*), visible phenotypic variation (*P*_*normal*_), and the accumulation of cryptic variations (*P*_*cryptic*_) are shown. The *α* levels were adjusted to control for multiple comparisons using the false discovery rate (FDR) procedure.

### Effects of selective pressure and the number of within-generation environments on the accumulation of cryptic variations

**Figure 6:**
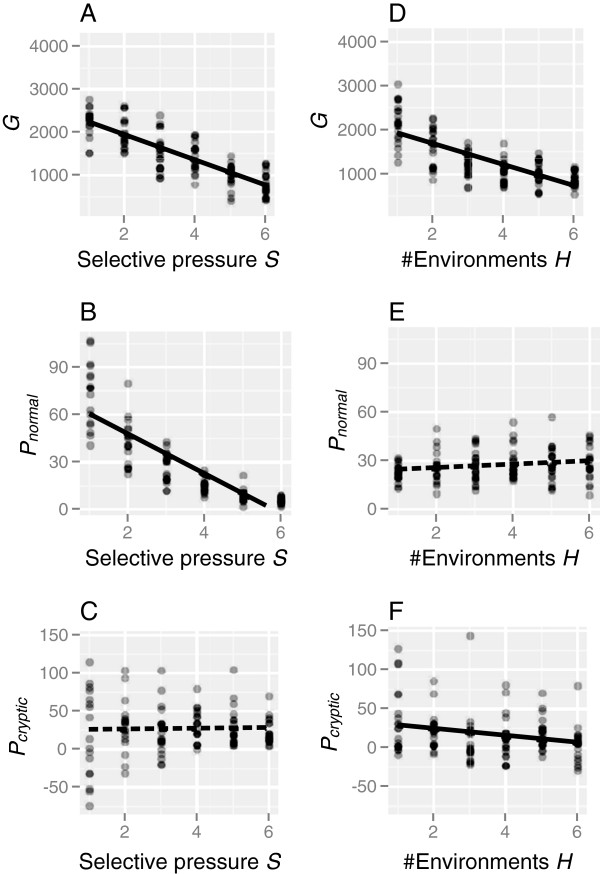
**Relationships between variations in a population and environmental parameters.** Simulations were repeated 20 times for each parameter value, and each dot represents a simulation run. Regression lines were drawn on the basis of the linear regression statistics shown in Table 2; solid and dashed lines denote significant and non-significant correlations, respectively.

The effects of parameters related to environmental factors, the strength of stabilizing selection (*S*), and the number of environments that individuals experienced within their lifetimes (*H*) were examined (Table 2). Increased strength of the stabilizing selection decreased *G* and *P*_*normal*_ but did not affect *P*_*cryptic*_ (Figure 6A-C). The numbers of genetic variations (*G*) and cryptic variations (*P*_*cryptic*_) significantly decreased with the number of environments that individuals experienced within their lifetimes, whereas the visible phenotypic variations (*P*_*normal*_) were not affected (Figure 6D-F).

### Effects of altered parameters on network characteristics

The effects of varying the parameter values on the network properties of evolved populations were also analyzed Additional file 2: (Table S2). As expected, the mutation rate (*μ*) and population size (*N*) did not affect the network properties of the evolved populations. The network size significantly increased with increasing numbers of genes (*V*_*genome*_) and *cis*-regulatory elements (*L*). The increasing number of genes (*V*_*genome*_) caused an increase in network order and a decrease in density, whereas the increasing number of *cis*-regulatory elements (*L*) caused an increase in network density.

**Figure 7:**
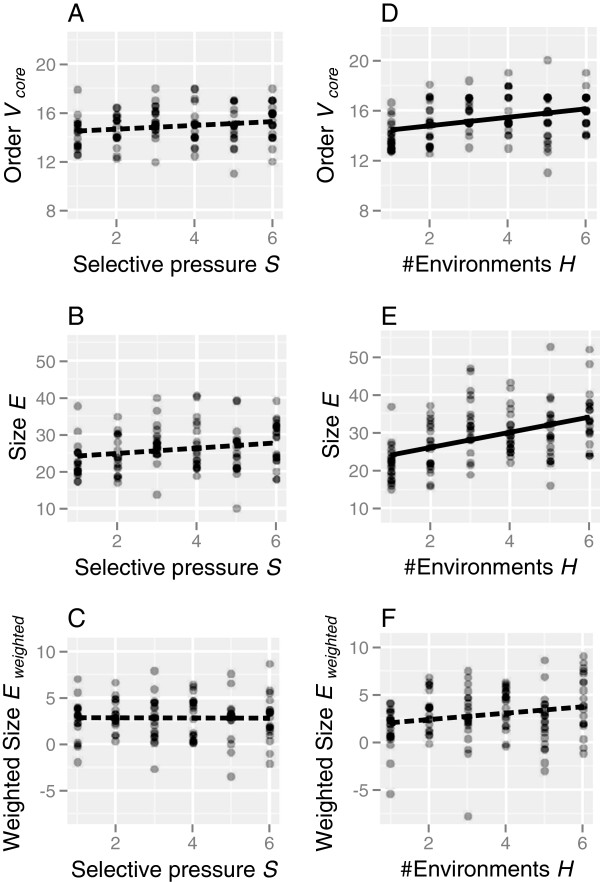
**Relationships between GRN features and environmental parameters.** Simulations were repeated 20 times for each parameter value, and each dot represents a population mean of a simulation run. Regression lines were drawn on the basis of the linear regression statistics shown in Table S2; solid and dashed lines denote significant and non-significant correlations, respectively.

The strength of stabilizing selection (*S*) did not affect the network properties of the evolved populations (Figure 7A-C). The order, size, and density of the evolved GRNs were significantly increased with exposure to increasing numbers of environments within a lifetime (*H*; Figure 7D-F). In other words, larger GRNs with higher connectivity tended to evolve in heterogeneous environments.

## Discussion

### Factors affecting the number of cryptic variations

The accumulation and release of CGVs were predicted to be the outcome of epistasis and genotype–environment interactions by analytical population genetic modeling [19]. Hermisson and Wagner (2004) assumed sexual reproduction and argued that the sex dependence of allelic effects is an important mechanism for conditional neutrality. In the context of a recent categorization [16], their model corresponds to “post-mutation evolvability under high recombination,” whereas ours corresponds to “pre-mutation evolvability under low recombination.” Here we showed that such epistatic behavior could be produced as a generic feature of GRNs even without sexual reproduction and without an explicit coefficient for hidden variation as expected from previous stochastic models [29,34,53-56]. The present study used individual-based models with explicit implementation of a population of GRNs that produce phenotypes responding to environmental stimuli, and our findings indicate that the cryptic phenotypic variations unveiled by a novel environmental stimulus can accumulate and be maintained in evolved populations. The signals induced by a novel environmental stimulus probably initiated different cascades of gene interactions, which led to novel phenotypic variations due to standing genetic variations that were invisible under normal circumstances.

In the present study, we used two methods to examine the effects of factors that affect the number of CGVs. First, because genetic variations and some network properties varied among the simulation replicates for the same parameter set values, we examined the effects of varying values of these properties. Second, we altered the parameter values and examined the effects of varying parameter values on the CGVs. In our simulations, the number of genes, *V*_*genome*_, was not assumed to be altered during the evolutionary process; therefore, the effect of *V*_*genome*_ could be examined by altering the initial values of the parameters.

The number of CGVs accumulated in each of the populations increased with increases in the total numbers of genetic polymorphisms (Figure 4H) when the simulation replicates with the default parameters were analyzed. This is reasonable because the more polymorphic genotypes a population has, the more diverse phenotypes it is supposed to produce. This finding was also supported by the results of the simulations where only the population size and mutation rate were altered (Table 2). In addition, increasing the network order by altering the number of genes increased the number of total and cryptic genetic variations even under conditions where the mutation rate per individual (not per gene) was kept constant.

Under the condition of a fixed number of genes, the number of CGVs increased with increasing the weighted size of the GRNs (Figure 4C). The weighted size of a GRN was expected to increase when increasing the order, size, and ratio of the positive edges. In the second analysis, increasing the number of genes caused increases in the order and size of the GRNs followed by an increase in the number of CGVs. In addition, it was confirmed that increasing the ratio of positive edges by altering the positive edge bias caused increases in the weighted size and the number of CGVs in the GRNs. The genetic difference among networks with large weighted sizes may have been amplified through genetic interactions and resulted in larger differences in expression outcomes, which would have contributed to the emergence of CGVs.

These findings indicate that the number of genes could affect the number of CGVs in two ways. First, a population of GRNs with a large number of genes would be able to accumulate a larger number of genetic variations. Second, a higher network order would have a larger weighted size, which may contribute to the emergence of more phenotypic variations. Thus, we propose that the network order (i.e., the number of genes) is the most important factor in facilitating the accumulation and release of CGVs.

### Effect of strength of selection and environmental variations

The accumulation of CGVs was affected by the strength of the selective pressure and the number of environments that the individuals experienced during their lifetime. Increasing the strength of selection and the number of environments decreased the genetic variation to a similar extent; however, the effects of these two environmental factors on the number of CGVs differed. This indicates that the quality of accumulated genetic variations may differ depending on the ancestral environments that the populations experienced through the generations.

We predicted that strong selection usually purges genetic variation within populations; therefore, increasing the strength of stabilizing selection decreases the number of genetic and phenotypic variations. The present findings showed that this prediction was only true for visible variations and not for cryptic variations. The number of CGVs increased despite the strong selective pressure. Not all mutations in the GRNs led to phenotypic differences in a normal environment. The mutations that caused phenotypically visible differences were purged by strong pressure, whereas those that did not affect the phenotype were free from selection and accumulated in populations through mutation-drift balance. Therefore, the genetic variations that did not contribute to the phenotypes in the normal environments were preserved in the populations regardless of the strength of the selective pressure.

The number of environments an individual was exposed to during a lifetime also decreased the accumulation of genetic variations. Here, the individuals were required to express different phenotypes in response to different environmental signals experienced during their lifetimes using appropriate regulatory pathways. When an individual experienced a larger number of environments, more of the GRN should be involved in producing phenotypic differences than that in normal environments. Therefore, exposure to a larger number of different environments would probably decrease the number of mutations accumulated through genetic drift. In other words, populations with high plasticity may have less evolvability than those with less plasticity, which would result in less phenotypic diversity in response to novel environments. An experimental study indicated that populations that evolved in rapidly changing environments may result in genotypes with high levels of pleiotropy and historical constraints, which in tern could impede the future evolution [57]. This finding indicates that there may be intrinsic genetic costs associated with adaptation to variable environments [58-60]. These costs differ from other types of costs associated with plasticity because they cannot be directly detected by negative correlations between individual fitness and plasticity but can instead emerge as reduced genetic variability on an evolutionary timescale. However, some predictions [54-56] contradict this. One possible reason for this discrepancy is a difference in focus. Espinosa-Soto et al. [54] showed that perturbing the initial state of each GRN every generation shortened the time required to discover the genotype of an arbitrary novel phenotype. Fierst [55] measured the evolutionary variability of genetic systems toward novel phenotypic optima without changing the environmental cue. Draghi and Whitlock [56] emphasized the directionality of plasticity and evolvability. Here we investigated the quantitative tendencies of genetic and phenotypic variations and detected a negative effect of environmental heterogeneity on the number of variations present in a population.

On the other hand, environmental heterogeneity would be expected to have positive effects on the number of CGVs in the light of GRN evolution. The more heterogeneous the environment that individuals experienced during their lifetimes, the larger and denser the GRNs tended to become Additional file 2: (Table S2). This was probably because only small parts of the GRNs were used in low heterogeneity environments and then unused genes were then allowed to mutate and become disconnected from the GRNs without affecting their fitness. In other words, populations that evolve in static environments over long periods may lose the ability to sense and respond to the environment, and would therefore show less revealed phenotypic variations in novel environments. A previous theoretical study [36] showed that the evolution of complex GRNs could be remarkably promoted by the fixation of beneficial gene duplications under randomly fluctuating environmental conditions and that such GRNs tend to exhibit high mutational robustness and evolvability. Furthermore, they showed that large and complex GRNs could not be evolved in cyclically fluctuating environments. This indicates that predictable variable environments, including the heterogeneous environments that we used here, cannot promote the evolution of higher evolvability as well as of larger numbers of CGVs. Therefore, interactions with variable environments in which higher plasticity has evolved may promote the accumulation of CGVs by facilitating the evolution of larger GRNs in some cases. In turn, the expansion of GRNs could facilitate evolutionary adaptation to novel environments and niche construction. Thus, we propose that the interaction between GRNs and variable environments could be a sustainable source of evolvability.

### Acquisition of novel traits promoted by cryptic genetic variations in GRNs

It is believed that CGVs contribute to evolutionary responses to environmental changes and to long-term selection by providing phenotypic variations in response to changes in the background [8,9,61]. Furthermore, some researchers argue that CGVs also promote the acquisition of novel traits because of their ability to accumulate and release multiple mutations in individuals and populations [62,63].

Several studies support these arguments. For example, the black mutant strain of Manduca sexta, which was originally green, showed variations in thermosensitivity where some larvae turned green and the others stayed black in response to heat shock over a specific period [64]. The authors of that study also artificially selected these variations and successfully established two lines: one for sensitivity and the other for insensitivity. CGVs that accumulate during strong stabilizing selection could contribute to phenotypic evolution in this manner.

In addition, a previous model study [34] predicted that GRNs are robust and evolvable enough to cover the broad genotypic space and to reach genotypes that produce novel phenotypes through recurrent neutral mutations. Our individual-based model revealed that CGVs could accumulate and produce phenotypic diversity to contribute to evolvability in the context of population genetic processes. It has been proposed that novel phenotypes do not necessarily require new genes. Rather, changes in the expression patterns of existing genes are important for phenotypic evolution [65-68]. Further simulations with properly defined novel phenotypes will help elucidate the evolution of novelty.

## Conclusions

We constructed an individual-based model of GRNs that controlled gene expression in response to environmental stimuli. The model facilitated the analysis of network properties in the context of population genetics. It showed that populations of GRNs accumulate and release cryptic variations, the number of which varies depending on the properties of the GRNs and the environments to which they have been subjected across the generations. Our findings indicate that the expansion of GRNs and adaptation to novel environments are mutually facilitating, resulting in a sustainable sources of evolvability. This study thus provides important insight into the origins of biological diversity and complexity.

## Abbreviations

CGV: Cryptic genetic variation.

## Authors’ contributions

WMI conceived the study, constructed the computer simulations, performed the analyses, interpreted the results, and drafted the manuscript. MET contributed the program codes. MK interpreted the results and helped to draft the manuscript. All authors read and approved the final manuscript.

## Supplementary Material

Additional file 1 **Table S1.** Statistics for nested model comparisons. Model selection statistics for the effects of GRN features on the number of cryptic genetic variations in a population. Among all possible combinations, only the models for which the AIC values differed from that of the null model by ≥2 are shown. Note that all these models include the weighted size of the GRN (*E*_*weighted*_) as an explanatory variable. Click here for file

Additional file 2 **Table S2.** The effects of each parameter on the evolution of GRNs. The effects of population size (*N*), mutation rate (*μ*), number of genes (*V*_*genome*_), number of *cis*-elements (*L*), positive edge bias (*A*), strength of selection (*S*), and number of environments (*H*) on network properties are shown. The *α* levels were adjusted to control for multiple comparisons using the false discovery rate (FDR) procedure. Click here for file
